# Coinfection With Influenza A Virus and *Klebsiella oxytoca*: An Underrecognized Impact on Host Resistance and Tolerance to Pulmonary Infections

**DOI:** 10.3389/fimmu.2018.02377

**Published:** 2018-10-29

**Authors:** Kayla M. Lee, Jenna Morris-Love, Damien J. Cabral, Peter Belenky, Steven M. Opal, Amanda M. Jamieson

**Affiliations:** ^1^Department of Molecular Microbiology and Immunology, Brown University, Providence, RI, United States; ^2^Department of Molecular Biology, Cell Biology and Biochemistry, Brown University, Providence, RI, United States; ^3^Department of Medicine, Warren Alpert School of Medicine, Brown University, Providence, RI, United States

**Keywords:** coinfection, influenza A virus, *Klebsiella oxytoca*, disease tolerance, pulmonary infection

## Abstract

Pneumonia is a world health problem and a leading cause of death, particularly affecting children and the elderly (1, 2). Bacterial pneumonia following infection with influenza A virus (IAV) is associated with increased morbidity and mortality but the mechanisms behind this phenomenon are not yet well-defined (3). Host resistance and tolerance are two processes essential for host survival during infection. Resistance is the host's ability to clear a pathogen while tolerance is the host's ability to overcome the impact of the pathogen as well as the host response to infection (4–8). Some studies have shown that IAV infection suppresses the immune response, leading to overwhelming bacterial loads (9–13). Other studies have shown that some IAV/bacterial coinfections cause alterations in tolerance mechanisms such as tissue resilience (14–16). In a recent analysis of nasopharyngeal swabs from patients hospitalized during the 2013–2014 influenza season, we have found that a significant proportion of IAV-infected patients were also colonized with *Klebsiella oxytoca*, a gram-negative bacteria known to be an opportunistic pathogen in a variety of diseases (17). Mice that were infected with *K. oxytoca* following IAV infection demonstrated decreased survival and significant weight loss when compared to mice infected with either single pathogen. Using this model, we found that IAV/*K. oxytoca* coinfection of the lung is characterized by an exaggerated inflammatory immune response. We observed early inflammatory cytokine and chemokine production, which in turn resulted in massive infiltration of neutrophils and inflammatory monocytes. Despite this swift response, the pulmonary pathogen burden in coinfected mice was similar to singly-infected animals, albeit with a slight delay in bacterial clearance. In addition, during coinfection we observed a shift in pulmonary macrophages toward an inflammatory and away from a tissue reparative phenotype. Interestingly, there was only a small increase in tissue damage in coinfected lungs as compared to either single infection. Our results indicate that during pulmonary coinfection a combination of seemingly modest defects in both host resistance and tolerance may act synergistically to cause worsened outcomes for the host. Given the prevalence of *K. oxytoca* detected in human IAV patients, these dysfunctional tolerance and resistance mechanisms may play an important role in the response of patients to IAV.

## Introduction

During the influenza season an average of 20% of the human population is infected, with this percentage varying from year to year depending on the virulence of the strains circulating that season (18). Secondary bacterial pneumonia following influenza A virus (IAV) infection is a serious complication whose prevalence and severity correlates with the virulence of the influenza strain (3, 19). On average, 0.5% of previously healthy, young individuals and 2.5% of elderly or immunocompromised patients that contract IAV have bacterial coinfections; however, during times of influenza pandemic these numbers climb even higher and in the 1918 influenza virus pandemic up to 6.1% of all patients with IAV were thought to have secondary bacterial infections (20). In 1918, prior to the use of antibiotics, autopsies confirmed the presence of bacteria in up to 95% of fatalities (3, 21). In the 2009 pandemic between 18 and 34% of IAV patients in the ICU had a bacterial coinfection and up to 55% of fatalities were associated with bacterial coinfection (21, 22).

The bacteria that are most commonly implicated in coinfection with IAV are *Streptococcus pneumoniae, Staphylococcus aureus, Haemophilus influenzae, Legionella pneumophila, Pseudomonas* species, and *Klebsiella* species (18). The development and use of antibiotic treatment has increased the prevalence of antibiotic-resistant bacterial strains, such as methicillin-resistant *S. aureus* (MRSA), implicated in coinfection as well (18). However, due to the significant overlap in symptoms of pneumonia caused by influenza virus infection alone vs. coinfection, diagnoses of coinfection are difficult to make and often antibiotics are inappropriately administered (18). With the growing concern about antibiotic- and antiviral-resistant pathogens, it is clear that more emphasis needs to be placed on finding alternative therapies to treat coinfection. Currently the IAV vaccine, while it does impart some protection and can decrease the severity of symptoms, has variable effectiveness due to the antigen drift that occurs each season (3). Even with advances in treatments against pathogens such as vaccines, antivirals, and antibiotics, bacterial coinfection still represents a major threat to human health (23, 24).

Host resistance and host tolerance are two important factors that can determine the outcome of a patient following infection (4–8). The ability to successfully detect and eliminate pathogens is called host resistance while the ability to overcome the damaging effects caused by the pathogen and the immune response to that pathogen is known as host disease tolerance or resilience. If the host lacks either one of these properties, it becomes susceptible to infection (4–8). Bacterial coinfections can cause increased mortality due to alterations in either resistance or tolerance; for example, *S. pneumoniae* coinfections are characterized by an increased bacterial burden which overwhelms the host, whereas *L. pneumophila* coinfections cause mortality through a significant amount of tissue damage without an increase in pathogen burden (14, 25). IAV/*S. pneumoniae* coinfection may be an example of decreased resistance leading to alterations in tolerance as there is also increased tissue damage, but given the overwhelming bacterial burden it is challenging to separate out these two mechanisms (26). Because each type of IAV/bacterial coinfection can cause mortality through different mechanisms, it is important to study them individually to uncover the best way to treat them.

Up until now, the majority of studies on IAV/bacterial coinfection have focused on *S. pneumoniae* and *S. aureus*. While these are two of the most prevalent bacteria in coinfections with IAV, there are many other bacteria that have been vastly understudied (18). This includes *Klebsiella spp* which are gram-negative, opportunistic pathogens responsible for between 3 and 7% of all nosocomial infections including UTIs, septicemia, and pneumonia (17). Pneumonia caused by *Klebsiella spp* has up to a 50% fatality rate and the emergence of multi-drug-resistant strains has made it increasingly difficult to treat (17, 27). Among this genus is *Klebsiella oxytoca* which is a pathobiont in the human microbiome and an underrecognized contributor to hospital-acquired pneumonia in immunocompromised patients (28). The involvement of *K. oxytoca* in bacterial coinfections with IAV has of yet been unclear. A recent study from Gao et al. identified the presence of *K. oxytoca* in one H7N9 patient from a cohort in China in 2013 (29). Data presented here indicates that its prevalence is potentially underestimated and therefore should be a target for further study. Our lab has detected an increased presence of *K. oxytoca* in nasopharyngeal swabs from patients who tested positive for IAV in Rhode Island during the 2013–2014 influenza season and this finding prompted us to investigate the immunological responses that occur during coinfection with IAV and *K. oxytoca*.

To study the pathogenesis of IAV/*K. oxytoca* coinfection we developed a mouse model in which we observed increased mortality in coinfected animals compared to singly-infected controls. Within our model system, we saw a heightened inflammatory response following coinfection but despite an increase in immune cell infiltrate, there was a delay in bacterial clearance. In addition, we observed an increase in tissue damage as a result of coinfection, perhaps caused by a shift in macrophage polarization away from a tissue reparative phenotype. As such, this model is an excellent vehicle to study host resistance and tolerance since both are impacted as a result of coinfection. Our work studying coinfections with IAV and the previously underrecognized *K. oxytoca* highlight the complex relationship between host resistance and tolerance and suggest the need for further study of these systems.

## Results

### Detection of *Klebsiella oxytoca* in nasopharyngeal swabs from IAV patients

While pneumonia caused by *K. oxytoca* has recently been reported in one IAV-infected patient (29), the overall prevalence of *K. oxytoca* among IAV patients is as yet unknown. In order to investigate this, we looked for the presence of *K. oxytoca* in nasopharyngeal swabs from a cohort of patients admitted to the Memorial Hospital in Rhode Island during the influenza season of 2013–2014. Our findings show that among patients that tested positive for IAV there was a significantly higher proportion that also tested positive for *K. oxytoca* (14.00%) compared to those patients that tested negative for IAV (3.88%), implying that infection with IAV increases susceptibility to *K. oxytoca* colonization (Table 1). While these data show a clear association of IAV patients with *K. oxytoca*, it is unknown whether these patients had an active secondary infection with *K. oxytoca* or whether IAV infection enhances susceptibility to *K. oxytoca* colonization without causing infection. We also looked for the presence of *S. pneumoniae* in this cohort and found a similar trend to *K. oxytoca* in which a higher percentage of IAV-positive patients also tested positive for *S. pneumoniae* (20.00%) compared to IAV-negative patients (8.53%) (Table 1). *S. pneumoniae* is commonly implicated in secondary bacterial infections with IAV and is known to cause increased morbidity and mortality in these cases. Our findings showed similar patterns in the association of IAV patients with *K. oxytoca* as *S. pneumoniae*, which led us to question whether *K. oxytoca* is likewise able to alter host responses during coinfection with IAV to cause worsened outcomes.

**Table 1:** Influenza patients are more susceptible to bacterial colonization by *Streptococcus pneumoniae* and *Klebsiella oxytoca*.

	**Influenza–**	**Influenza+**
*S. pneumoniae –*	91.47%	80.00%
*S. pneumoniae +*	8.53%	20.00%
Total number of patients	129	50
*K. oxytoca –*	96.12%	86.00%
*K. oxytoca +*	3.88%	14.00%
Total number of patients	129	50

*Nasopharyngeal swabs from a total of 179 patients were tested for the presence of influenza as well as S. pneumoniae and K. oxytoca. Of the 50 patients that tested positive for influenza, 10 (20.00%) also tested positive for S. pneumoniae and 7 (14.00%) tested positive for K. oxytoca. In contrast, of the 129 patients that tested negative for influenza, only 11 (8.53%) also tested positive for S. pneumoniae and 5 (3.88%) tested positive for K. oxytoca. These results indicate that infection with influenza leads to an increased association with several bacterial species. Statistics were calculated by Fisher's exact test with P = 0.0403 for S. pneumoniae and P = 0.0391 for K. oxytoca*.

### Coinfected mice exhibit increased inflammation and cellular infiltrate early after bacterial infection

**Figure 1:**
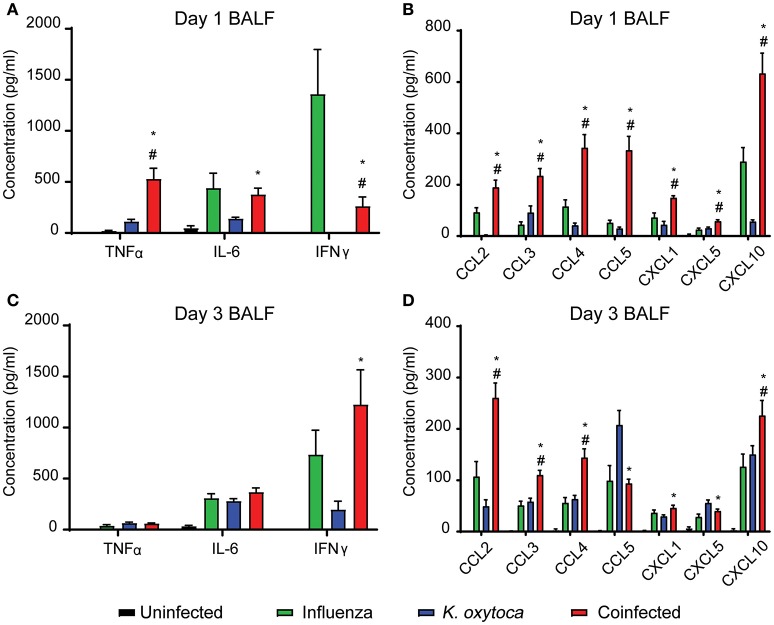
Coinfected mice exhibit increased inflammation early after bacterial infection. Protein concentrations of a panel of cytokines and chemokines were measured in the bronchoalveolar lavage fluid (BALF) of mice on days 1 and 3 following *K. oxytoca* infection (days 8 and 10 following IAV). Day 1 cytokine levels **(A)** and chemokine levels **(B)** are depicted for all 4 groups. Day 3 cytokine **(C)** and chemokine **(D)** levels are depicted for all 4 groups. # denotes *P* ≤ 0.05 between coinfected and IAV groups. ^*^ denotes *P* ≤ 0.05 between coinfected and *K. oxytoca* groups. Data were analyzed with ANOVA followed by Tukey's multiple comparison tests. Error bars represent SEM. Data are combined from at least four independent experiments with at least four mice per group.

In order to investigate the host response to infection during IAV and *K. oxytoca* coinfection, we developed a mouse model in which a sublethal dose of IAV was administered followed by a sublethal dose of *K. oxytoca* 5 days after IAV. First, we assessed whether coinfection induced changes to the inflammatory response early following bacterial infection as has been observed in other coinfection models (9, 25, 30–32). We measured the concentrations of a panel of inflammatory cytokines and chemokines in the bronchoalveolar lavage fluid (BALF) including IL-6, TNFα, IFNγ, CCL2, CCL3, CCL4, CCL5, CXCL1, CXCL5, and CXCL10 (Figure 1). These cytokines and chemokines are essential in the innate immune response to both bacteria and viruses. On day 1 post-coinfection, there was an early increase in the production of TNFα during coinfection that was not observed in either singly-infected group whereas levels of IL-6 were equal between IAV-infected and coinfected groups (Figure 1A). We also saw a significant amplification in the production of all chemokines measured during coinfection compared to any other group (Figure 1B). The only reduction in cytokine levels that we observed in the coinfection was in IFNγ; however, IFNγ during coinfection was still significantly increased over the group infected with *K. oxytoca* alone (Figure 1A). By day 3, IFNγ levels in the coinfected lungs overtook those seen in the singly-infected groups and reached the level seen in IAV-infected lungs on day 1, indicating a delay in the kinetics of IFNγ during coinfection (Figure 1C). At day 3 post-coinfection, most chemokines remained elevated in the coinfected lungs, although these levels were decreased overall from day 1 with the exception of CCL2 (Figure 1D). Only CCL5 and CXCL5 concentrations were higher in the group with *K. oxytoca* alone than coinfection (Figure 1D). These results indicate that the coinfected lung was able to sense the presence of both pathogens and increase production of multiple inflammatory signals in response. This also shows that there is an early, robust response on day 1 that tapers but remains elevated by day 3 post-coinfection.

**Figure 2:**
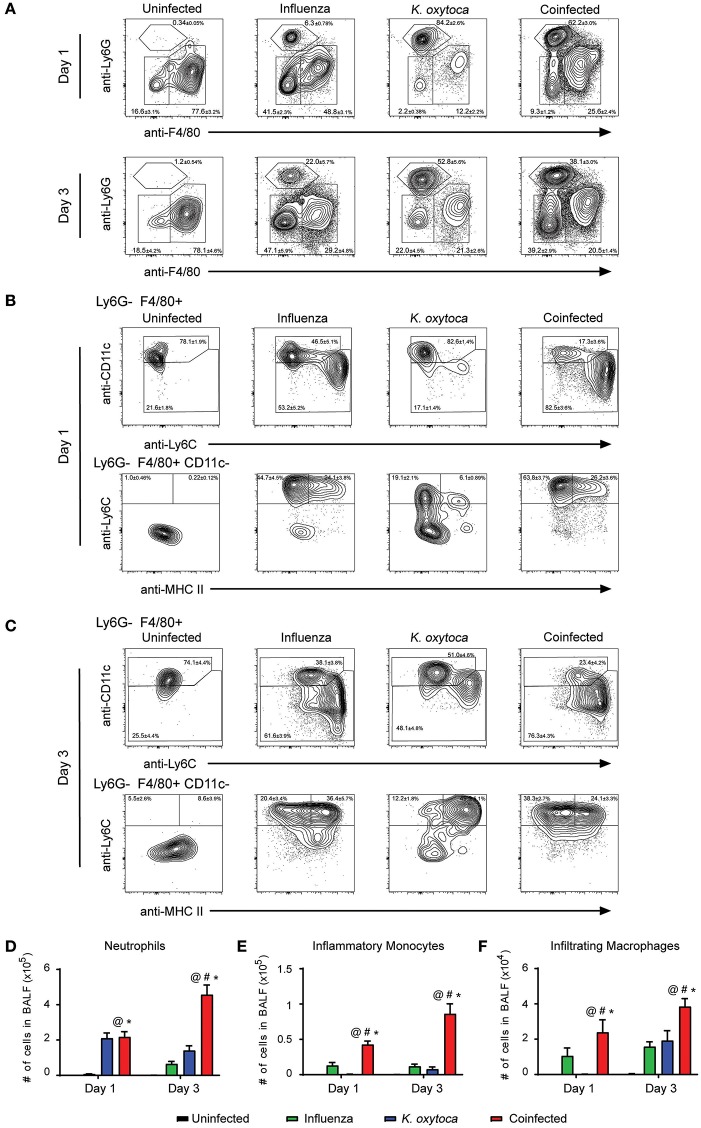
Immune cell infiltrate is increased in the BALF of coinfected mice. Innate immune cell populations were quantified in the BALF of mice on days 1 and 3 post-*K. oxytoca* infection. We identified neutrophils as Ly6G^+^F480^−^ cells and separated the F480^+^ population according to expression of CD11c with alveolar and repopulating macrophages identified as Ly6G^−^F480^+^CD11c^+^ cells. The CD11c^−^ population was further separated by expression of Ly6C and MHC II with inflammatory monocytes identified as Ly6G^−^F480^+^CD11c^−^Ly6C^+^MHC II^−^ cells and infiltrating macrophages identified as Ly6G^−^F480^+^CD11c^−^Ly6C^+^MHC II^+^ cells **(A–C)**. The total number of neutrophils **(D)**, inflammatory monocytes **(E)**, and infiltrating macrophages **(F)** from days 1 and 3 were calculated according to their percentages of the total population and the total number of cells collected in each BALF. @ denotes *P* ≤ 0.05 between coinfected and uninfected groups. # denotes *P* ≤ 0.05 between coinfected and influenza groups. * denotes *P* ≤ 0.05 between coinfected and *K. oxytoca* groups. Data were analyzed with ANOVA followed by Tukey's multiple comparison tests. Error bars represent SEM. Data are combined from at least four independent experiments with at least four mice per group.

After observing that coinfection with IAV/*K. oxytoca* was characterized by a significant amplification of inflammatory cytokines and chemokines, we next investigated how this inflammatory milieu might affect which innate immune cells traffic to the lung and the magnitude of their recruitment as compared to either single viral or bacterial infections. Using a flow cytometric panel of markers to identify different innate immune cell subsets, we identified Ly6G^−^F480^+^CD11c^+^ macrophages which include both alveolar macrophages and macrophages that upregulate CD11c as they infiltrate the lungs, Ly6G^−^F480^+^CD11c^−^Ly6C^+^MHC II^−^ inflammatory monocytes, Ly6G^−^F480^+^CD11c^−^Ly6C^+^MHC II^+^ infiltrating inflammatory macrophages, and Ly6G^+^F480^−^ neutrophils (Figures 2A–C). For identification of lung macrophages, we compared expression of CD11c and Siglec-F (known alveolar macrophage markers) and found that on day 1 post-coinfection, all macrophages that expressed CD11c also expressed Siglec-F and were therefore all alveolar macrophages, but on day 3 there was a percentage of CD11c^+^ cells in the coinfected group that did not express Siglec-F, potentially representing a population of infiltrating macrophages that upregulate CD11c as they repopulate the lungs following infection (Supplemental Figure 2) (33). For complete description of gating strategies see Supplemental Figures 1, 2.

We first examined cell subsets in the BALF (Figure 2). The Ly6G^−^F480^+^CD11c^+^ macrophage population did not show any notable changes in number throughout the course of any single or dual infection. However, neutrophil numbers increased dramatically during coinfection. One day post-coinfection, coinfected lungs showed similar neutrophil numbers to *K. oxytoca*-infected lungs; however, by 3 days post-coinfection, neutrophil numbers from coinfected lungs were increased greater than three-fold over *K. oxytoca*-infected animals and greater than seven-fold over IAV-infected animals (Figures 2A,D). This delayed but significant increase in the recruitment of neutrophils was likely in part caused by the early induction of many chemokines that recruit neutrophils, such as CXCL1 and CXCL5, at day 1 post-coinfection (Figure 1B). In addition, both the inflammatory monocyte and infiltrating macrophage populations expanded significantly during coinfection at both days 1 and 3 post-coinfection in the BALF when compared to other groups (Figures 2B,C,E,F).

**Figure 3:**
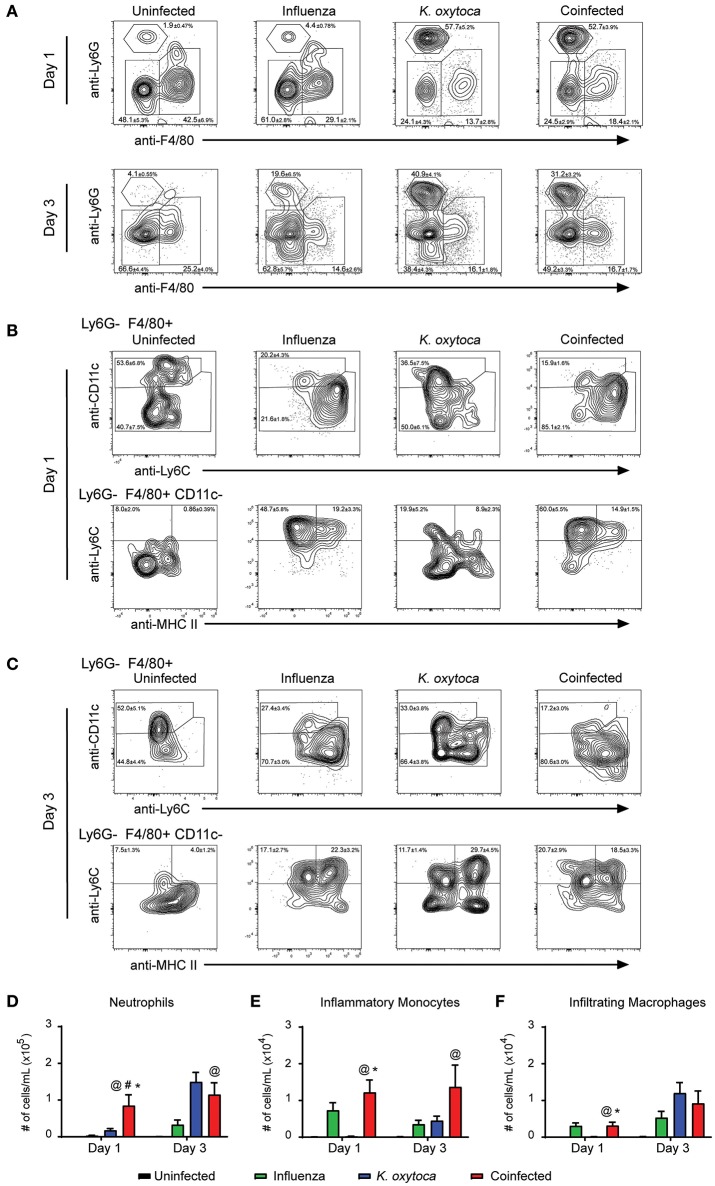
Immune cell infiltrate is increased in the lungs of coinfected mice. Innate immune cell populations were quantified in the lungs of mice on days 1 and 3 post-*K. oxytoca* infection. We identified neutrophils as Ly6G^+^F480^−^ cells and separated the F480^+^ population according to expression of CD11c with alveolar macrophages and repopulating macrophages identified as Ly6G^−^F480^+^CD11c^+^ cells. The CD11c^−^ population was further separated by expression of Ly6C and MHC II with inflammatory monocytes identified as Ly6G^−^F480^+^CD11c^−^Ly6C^+^MHC II^−^ cells and infiltrating macrophages identified as Ly6G^−^F480^+^CD11c^−^Ly6C^+^MHC II^+^ cells **(A–C)**. The total number of neutrophils **(D)**, inflammatory monocytes **(E)**, and infiltrating macrophages **(F)** from days 1 and 3 were calculated according to their percentages of the total population and the total number of cells collected in each lung. @ denotes *P* ≤ 0.05 between coinfected and uninfected groups. # denotes *P* ≤ 0.05 between coinfected and influenza groups. * denotes *P* ≤ 0.05 between coinfected and *K. oxytoca* groups. Data were analyzed with ANOVA followed by Tukey's multiple comparison tests. Error bars represent SEM. Data are combined from at least four independent experiments with at least four mice per group.

We next determined changes in innate immune cells that infiltrated into the lung parenchyma during infection (Figure 3). In the lung tissue on day 1 post-coinfection, we observed a significantly greater number of neutrophils (Figures 3A,D), inflammatory monocytes (Figures 3B,E), and infiltrating macrophages (Figures 3C,F); however, by day 3 there were no significant differences in these populations between the infected groups which may be indicative of these cells trafficking through the lungs on day 1 to reach the alveolar space by day 3 (Figures 3C,D–F).

### Amplified innate immune responses do not enhance resistance in coinfected mice

**Figure 4:**
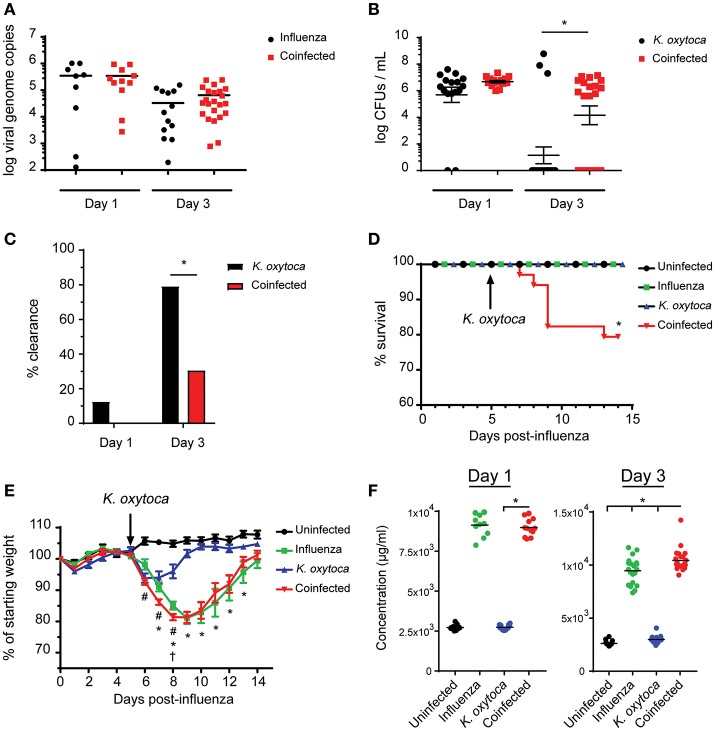
Coinfected mice exhibit increased morbidity and mortality as well as decreased resistance and tolerance. Viral burden as measured by viral genome copies in lung tissue on days 1 and 3 **(A)**. Bacterial burden as measured by colony-forming units (CFUs) in 1 mL of lung homogenate on days 1 and 3 **(B)**. Percentage of mice with no detectable bacteria in their lungs at day 1 and 3 **(C)**. Survival of naïve, singly-infected and coinfected mice was measured over the course of 2 weeks **(D)**. Weights were monitored during this time and are expressed as percentages of the starting weight of each mouse prior to infection **(E)**. Concentration of albumin in the BALF on days 1 and 3 post-*K. oxytoca* **(F)**. In **(E)** # denotes *P* ≤ 0.05 between coinfected and IAV groups while * denotes *P* ≤ 0.05 between coinfected and *K. oxytoca* groups. † denotes day at which coinfected mice begin to exhibit decreased survival. In **(C,D,F)** * denotes *P* ≤ 0.05 between indicated groups. Data were analyzed with ANOVA followed by Tukey's multiple comparison tests **(A,E,F)**, Mann-Whitney *U*-test on non-transformed data **(B)**, Fisher's exact test **(C)**, and log rank tests **(D)** where appropriate. Error bars represent SEM. Data are combined from at least three independent experiments with at least four mice per group.

Following our observations that coinfected lungs had a significantly heightened initial immune response compared to singly-infected lungs, we reasoned that this response was an attempt by the host to eliminate the dual pathogen burden. Therefore, we measured viral and bacterial loads in the lung throughout coinfection to determine if the immune response was able to effectively clear or control the pathogens. On day 1 post-coinfection, both viral and bacterial burdens were comparable between the coinfected and respective singly-infected groups (Figures 4A,B). However, by day 3 post-coinfection, although viral load remained equal between the coinfected and virally-infected groups, the coinfected mice displayed delayed bacterial clearance compared to the *K. oxytoca*-infected animals (Figures 4A–C). While most of the *K. oxytoca*-infected mice had cleared the bacteria by day 3, only 30% of coinfected mice had no detectable bacteria in their lungs (Figures 4B,C). Interestingly, despite delayed clearance during coinfection, all of the mice with detectable bacteria had similar bacterial burdens, regardless of whether or not they had a prior IAV infection. Since neither viral load nor bacterial colonies increased between days 1 and 3 in the coinfected lungs (Figures 4A,B), it appears that the immune response mounted was able to prevent both pathogens from overwhelming the host but had a defect in the early clearance of bacteria.

### Increased innate immune responses impact host tolerance during coinfection

To determine the effects of delayed clearance of *K. oxytoca* on the overall health of coinfected animals, we monitored the survival of coinfected mice as compared to singly-infected mice and observed that while all singly-infected groups were able to overcome infection, there was a significant decrease in survival of the coinfected animals starting just 3 days post-coinfection (Figure 4D). We also measured weights of these animals throughout an extended period of time following coinfection and found that all singly-infected animals lost weight but were able to recover back to their starting weights within 15 days of infection (Figure 4E). Coinfected mice exhibited a decrease in body weight comparable to the IAV-infected group but at an accelerated rate starting 1 day post-coinfection until several of the mice succumbed to disease (Figure 4E). With only a mild defect in host resistance as seen through delayed clearance of *K. oxytoca* in coinfected lungs, we questioned whether this phenomenon was responsible for the increased morbidity and mortality of these mice. Another possibility was that the worsened outcomes observed during coinfection were not as a result of uncontrolled pathogen replication but rather an inability of the host to tolerate the damage done by the massive immune response to the pathogens. To test this hypothesis, we measured the concentration of albumin in the BALF of infected mice as an indicator of vasculature leakage and therefore tissue damage in the lung (34). We found that as early as day 1 post-coinfection there was a significantly greater concentration of albumin in the lungs of coinfected mice as compared to singly-infected animals, and this observation was even more profound at day 3 post-coinfection (Figure 4F). Additionally, we looked at lactate dehydrogenase (LDH) release into the BALF as a measure of cell death and found that there was also increased LDH in coinfected BALF although this trend was not statistically significant (Supplemental Figure 2).

### Macrophage populations in coinfected mice are more pro-inflammatory compared to singly-infected mice

**Figure 5:**
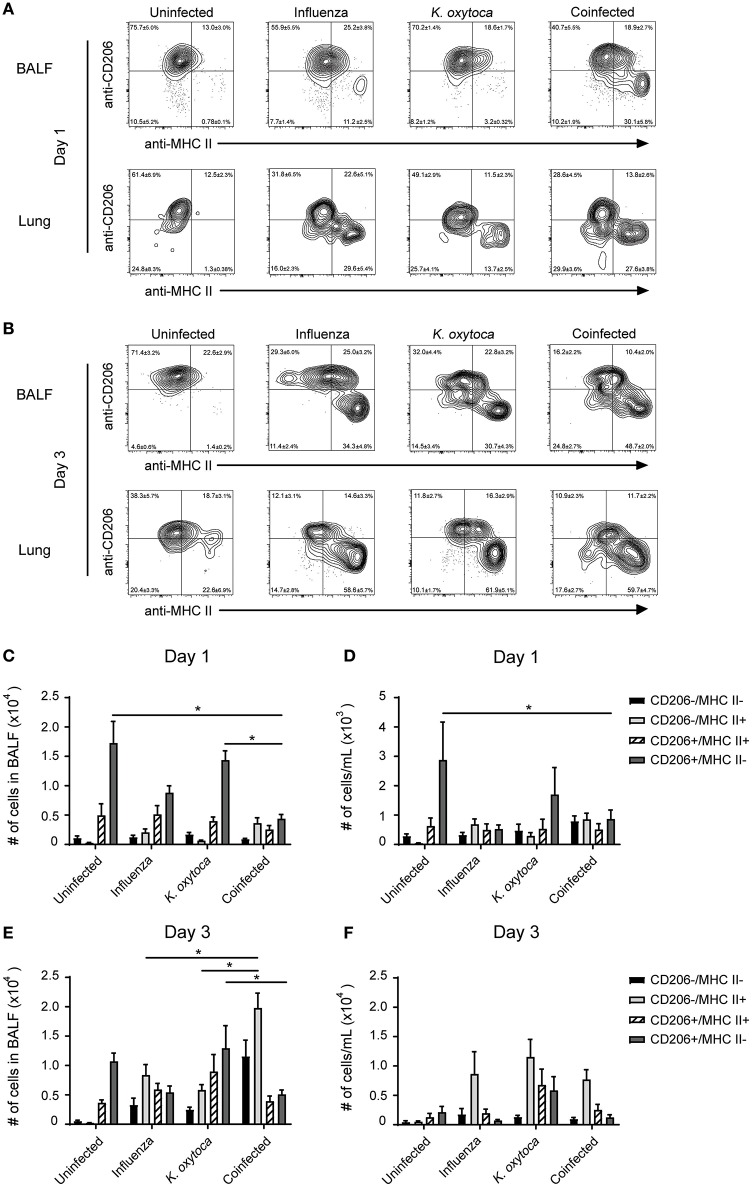
Macrophage populations in coinfected mice have increased MHC II and decreased CD206 compared to singly-infected mice. Ly6G^−^F480^+^CD11c^+^ macrophages in the BALF and lung tissue were analyzed for the expression of the MHC II and CD206 on days 1 and 3 post-*K. oxytoca* infection **(A,B)**. Macrophage subsets were classified as CD206^−^MHC II^+^, CD206^+^MHC II^+^, CD206^+^MHC II^−^, and CD206^−^MHC II^−^ and quantified in the BALF **(C,E)** and lung tissue **(D,F)** on days 1 and 3 post-*K. oxytoca*. * denotes *P* ≤ 0.05 between indicated groups. Data were analyzed with ANOVA followed by Tukey's multiple comparison tests. Error bars represent SEM. Data are combined from at least four independent experiments with at least four mice per group.

**Figure 6:**
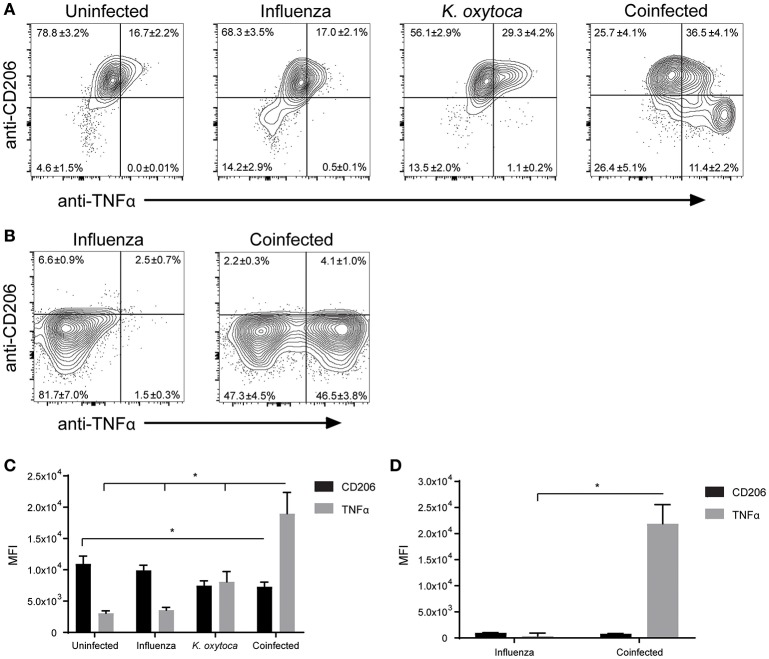
Macrophage populations in coinfected lungs produce more TNFα than during single infections. Ly6G^−^F480^+^CD11c^+^ **(A,C)** and Ly6G^−^F480^+^CD11c^−^ Ly6C^+^MHC II^+^ **(B,D)** macrophages in the BALF were analyzed on day 1 post-coinfection for expression of CD206 and production of TNFα as measured by mean fluorescent intensity (MFI). *denotes *P* ≤ 0.05 between indicated groups. Data were analyzed with ANOVA followed by Tukey's multiple comparison tests. Error bars represent SEM. Data are combined from at least four independent experiments with at least four mice per group.

After observing mild defects in both host resistance and tolerance during coinfection, we aimed to determine how the innate immune cells might be contributing to this progression of disease. Alveolar macrophages are the prominent cell type patrolling the lungs and are therefore often the first cells to encounter an invading pathogen (35). Alveolar macrophages will recognize and phagocytose pathogens, which triggers the release of a plethora of inflammatory cytokines and chemokines to attract other immune cells to the lung to help fight the infection (36). When they are not responding to pathogens, alveolar macrophages play an important role in maintaining homeostasis at steady state as well as mediating the return to homeostasis at the resolution of infection (35, 37, 38). They do this through the release of anti-inflammatory agents and factors that promote tissue repair, as well as by aiding in the catabolism of surfactant (35, 37–39). We hypothesized that during coinfection alveolar macrophages and macrophages that repopulate the lung following infection might play a role in shifting the balance toward pro-inflammatory and away from tissue repair processes. To test this, we explored changes in MHC II and CD206 expression on Ly6G^−^F480^+^CD11c^+^ macrophages as these are two markers of antigen presenting and tissue reparative phenotypes, respectively (35). We found that in a naïve lung, Ly6G^−^F480^+^CD11c^+^ macrophages were almost exclusively CD206^+^MHC II^−^ and at 1 day post-coinfection, Ly6G^−^F480^+^CD11c^+^ macrophages from the BALF and lungs of both singly-infected groups remained predominantly CD206^+^ with a small percentage expressing MHC II as well (Figures 5A,C,D). By day 3 post-coinfection the Ly6G^−^F480^+^CD11c^+^ macrophages from the IAV-infected BALF exhibited higher MHC II and lower CD206 expression (Figures 5B,E). Ly6G^−^F480^+^CD11c^+^ macrophages in the *K. oxytoca*-infected BALF at day 3 were still predominantly CD206^+^ (Figures 5B,E). In the lung at day 3, all infected groups showed an expansion in the MHC II^+^ population (Figures 5B,F). In coinfected BALF, there was a significant reduction in the number of CD206^+^ macrophages on day 1, while by day 3 there was also a significantly higher number of MHC II^+^ macrophages in the BALF (Figures 5C,E). Ly6G^−^F480^+^CD11c^+^ macrophages in the lung followed this trend but these results were not statistically significant. Additionally, we explored the capacity of the pulmonary macrophage populations to contribute to the inflammatory environment in the lung, particularly on day 1 post-coinfection (Figure 1). To do this, we looked for changes in TNFα production by Ly6G^−^F480^+^CD11c^+^ and Ly6G^−^F480^+^CD11c^−^ Ly6C^+^MHC II^+^ macrophages on day 1 post-coinfection (Figures 6A–D). On day 1 post-coinfection, the Ly6G^−^F480^+^CD11c^+^ macrophage population is made up entirely of alveolar macrophages, as indicated by their Siglec-F expression (Supplemental Figure 2) while the Ly6G^−^F480^+^CD11c^−^ Ly6C^+^MHC II^+^ macrophages are those that are infiltrating into the lung. The Ly6G^−^F480^+^CD11c^+^ alveolar macrophages exhibit a significant increase in their production of TNFα as compared to any singly-infected group (Figures 6A,C). On day 1, the Ly6G^−^F480^+^CD11c^−^ Ly6C^+^MHC II^+^ infiltrating macrophage population is absent in uninfected as well as *K. oxytoca*-infected BALF; however, comparing this population between IAV-infected and coinfected BALF, there is a significant increase in the production of TNFα during coinfection (Figures 6B,D). Also, it is notable that the Ly6G^−^F480^+^CD11c^−^Ly6C^+^MHC II^+^ infiltrating macrophages never express CD206 and are therefore not likely to be exhibiting a reparative phenotype (Figures 6B,D). These data indicate that both the Ly6G^−^F480^+^CD11c^+^ alveolar macrophages and the Ly6G^−^F480^+^CD11c^−^Ly6C^+^MHC II^+^ infiltrating macrophages are likely important contributors to the early, heightened inflammatory response during coinfection. Overall, these data showed that pulmonary macrophages from coinfected mice exhibit a more accelerated shift toward a pro-inflammatory phenotype when compared to singly-infected macrophages (Figures 5, 6). These results indicate that pulmonary macrophages play an important role in propagating the prolonged inflammatory response and delaying the reparative processes necessary to return to homeostasis following coinfection.

## Discussion

Thus far the vast majority of research on IAV/bacterial coinfections has focused on the bacteria *S. pneumoniae* or *S. aureus*; however, our data demonstrate that patients with IAV infection have a higher risk of association with the previously underrecognized bacteria *K. oxytoca*. Although it is unknown whether this increased association is due to increased susceptibility to infection with *K. oxytoca* or colonization without active infection, we decided to investigate the potential effects of coinfection with *K. oxytoca* since we saw similar trends of association as with IAV and *S. pneumoniae* which is known to result in severe coinfections with IAV. In addition, *K. oxytoca* was recently implicated in a study of IAV infection (29). Therefore, in order to explore the effects of coinfection with IAV and *K. oxytoca*, we developed a mouse model of IAV/*K. oxytoca* coinfection in which we observed that coinfected mice had increased morbidity and mortality when compared to singly-infected mice, as measured by survival and weight loss. As early as 1 day after coinfection, mice that had been previously infected with IAV had increased levels of several inflammatory cytokines and chemokines in the lung. The early induction of the inflammatory response that ultimately recruits innate immune cells is likely orchestrated by early pattern recognition receptor (PRR) signaling from pulmonary epithelial and endothelial cells (40–42). It is known that prior influenza infection can lead to a cytokine storm during secondary bacterial infection which begins with early pathogen-sensing by the epithelial cells and leads to massive infiltration of immune cells (40, 41). In conjunction with these findings, we observed an early increase in the levels of inflammatory cytokines day 1 post-coinfection in mice that were coinfected compared to mice with only one infection. In addition, following this increase in cytokine and chemokine levels in our model there was an increase in cellular infiltration into the lungs in coinfected mice by day 3 post-coinfection. Interestingly, this increased inflammatory infiltrate did not result in improved resistance to infection, as coinfected mice did not have a significant reduction in either viral or bacterial burden. Rather, 3 days after coinfection there was a decrease in the rate of clearance of *K. oxytoca* in mice that had an ongoing viral infection compared to those that did not. These results suggest a potential defect in the ability of the infiltrating immune cells to clear the bacteria. Although the increased inflammation in the lung during coinfection might have been expected to be accompanied by resultant lung damage, there was only a modest, albeit significant, increase in albumin in the BALF from coinfected mice, indicating a minor increase in vascular permeability compared to singly-infected lungs. It is unknown if this slight increase in vasculature leakage is enough to tip the balance toward increased morbidity and mortality or if there is another cause of decreased tolerance.

There are many examples of IAV/bacterial coinfection in which an overwhelming pathogen burden leads to a damaging inflammatory response (9–13). Some models of coinfection with IAV are characterized by an early, acute inflammatory response with increased production of TNFα among other cytokines. These models demonstrate that prior infection with IAV leads to bacterial overgrowth, tissue damage due to the heightened immune response, and ultimately decreased survival (9, 25, 30–32). Our model of IAV/*K. oxytoca* coinfection echoed the same types of inflammatory immune responses as have been seen in similar coinfection models. In contrast though, IAV/*K. oxytoca* coinfection did not result in increased bacterial or viral burden at early time points and bacterial burden was controlled despite a delay in clearance at later time points. These results indicate that decreased host resistance may not be solely responsible for decreased survival during coinfection with IAV/*K. oxytoca* and that perhaps there are other mechanisms which play a role in determining the outcome of the host during this particular coinfection.

Most studies of IAV/*S. pneumoniae* or IAV/*S. aureus* coinfections demonstrate that IAV infection suppresses the initial innate immune response to bacteria. IAV has been shown to impair neutrophil phagocytic activity and reactive oxygen species production which leads to increased susceptibility to bacterial infection (9, 43, 44). In addition, type I IFN production during IAV/bacterial coinfection has been shown to suppress CCL2, CXCL1, and CXCL2 levels and subsequently inhibit the recruitment of monocytes and neutrophils (45, 46). Type I IFN has also been demonstrated to suppress type 17 immunity and therefore increase susceptibility to secondary bacterial infection (47, 48). It has been reported that high levels of IFNγ suppress the expression of the scavenger receptor MARCO leading to decreased phagocytosis of bacteria by alveolar macrophages (49, 50). The only indication of immunosuppression in our model was a small drop in IFNγ levels 1 day after *K. oxytoca* infection in coinfected lungs compared to IAV infection alone; however, the levels in coinfected animals were higher than those seen in animals infected with bacteria alone. Also by day 3 post-coinfection, IFNγ levels in the coinfected mice increased and were higher than the other groups. Elevated IFNγ production at day 3 also corresponded to the time at which we observed a delay in bacterial clearance, which may indicate that IFNγ suppressed bacterial clearance at this later timepoint in the coinfected group. In contrast, *K. oxytoca*-infected mice produce less IFNγ and have no defect in bacterial clearance, supporting the notion that IFNγ may hinder clearance during coinfection.

Neutrophils are often essential in the response to bacterial infection, playing important roles in rapid clearance of the bacteria; however, their role during viral/bacterial coinfection has been less clear with some studies arguing their importance for tissue protection while others demonstrate more pathogenic roles (9, 44, 51). Our results showed that during IAV/*K. oxytoca* coinfection there was massive infiltration of neutrophils without a reduction in bacterial or viral load. One possible explanation for this outcome is that IAV infection impairs neutrophil phagocytic or bactericidal functions so that the neutrophils that are recruited to the lung following coinfection are less able to clear bacteria than naïve neutrophils. It has been shown that neutrophils from IAV-infected lungs have impaired phagocytosis as well as production of reactive oxygen species (ROS) and that increasing production of ROS by neutrophils and macrophages can reduce susceptibility to secondary bacterial infections (43, 44, 52). IAV infection has also been shown to diminish the production of granulocyte-colony stimulating factor (G-CSF), which is known to activate neutrophils, and administering this cytokine following IAV infection restores neutrophil bactericidal function (9). It has also been shown that during coinfection with IAV and *S. pneumoniae*, neutrophils produce excessive neutrophil extracellular traps (NETs) which cause extensive lung damage without any reduction in pathogen burden (53). In addition, neutrophils during IAV/bacterial coinfection have been shown to have accelerated apoptosis, which can cause tissue damage if not effectively cleared (54). This knowledge, coupled with our results of macrophages downregulating the efferocytic mannose receptor CD206, may point to a source of damage in our coinfection model (55). With this in mind, it is reasonable to suspect that neutrophils in IAV/*K. oxytoca* coinfected lungs are also dysfunctional, which might explain the delay in bacterial clearance and increase in tissue damage in these animals.

Lastly, many studies show that excess damage and the inability for the lung to return to homeostasis can cause decreased survival (14–16). Our data demonstrate that IAV/*K. oxytoca* coinfected lungs have increased vasculature leakage and tissue damage as indicated by a small but significant increase in albumin in the BALF as compared to IAV-infected lungs, whereas lungs infected with bacteria alone have virtually no lung damage. Although this increase in tissue damage is modest, it is possible that there is a threshold for the amount of damage that can be done to the lung and still allow for function and this small difference is a tipping point in morbidity and mortality during coinfection. One potential factor involved in the inadequate repair response following IAV/*K. oxytoca* coinfection is the shift in phenotype of pulmonary macrophages away from their native, homeostatic state. Macrophages are phenotypically flexible cells that can perform a variety of different roles depending on a number of environmental cues. On one side of the spectrum of macrophage phenotypes are M1 macrophages which have been defined by their role in recognizing certain bacterial and viral pathogens and generating inflammatory signals in response (55, 56). On the other side of the spectrum are M2 macrophages, which have been defined by their role in maintaining homeostasis through anti-inflammatory and tissue protective actions. However, these classifications are very broad and often do not accurately describe the nuanced states that macrophages can shift between (56). Alveolar macrophages normally play important roles during the resolution of infection to clear inflammatory agents and apoptotic cells and to remodel tissue (36–38, 55–57). However, pulmonary macrophage populations are also altered during inflammatory states, including infection and damage (58–60). The changes that occur to macrophage populations during IAV/bacterial coinfection have not been well studied. During coinfection with IAV/*K. oxytoca*, alveolar macrophages increase their production of TNFα and take on a more inflammatory phenotype. In addition, there is also a significant influx of macrophages and monocytes to the alveolar space during coinfection that tend to have a more inflammatory phenotype and likely also contribute to a shift in the macrophage population away from a reparative phenotype. Regardless of the origin of the pulmonary macrophages, whether they are alveolar macrophages or macrophages that have infiltrated and are repopulating the lung, it is evident that the population as a whole takes on a new role during coinfection that is directed more toward driving inflammation and is likely less conducive to repair. Several studies have demonstrated the important functions that macrophages play during lung infection. It has been demonstrated that the return to homeostasis mediated by macrophages is essential to survival of influenza infection and that mice suffer worsened outcomes in the absence of macrophages that promote tissue repair (61, 62). In addition, CD206 expression on macrophages is known to play important roles in removing potentially harmful extracellular enzymes generated during infection such as myeloperoxidases which are produced by neutrophils and cause tissue damage (63). CD206 is also an important mediator of phagocytosis of pathogens including influenza virus and *Klebsiella pneumoniae*, a bacterial species related to *K. oxytoca* (64–66). We observed downregulation of the mannose receptor CD206 in macrophages from coinfected lungs which could be indicative of a larger functional defect in important repair processes as well as recognition and clearance of pathogens. The overall shift in pulmonary macrophages toward a pro-inflammatory phenotype could be part of an ongoing effort to clear the bacteria that persists during coinfection. It is also likely that the influx of inflammatory cells is not only contributing to the damage done in the lung but also preventing the return to homeostasis by propagating and prolonging the inflammatory response. It is also possible that there are other factors that lead to decreased survival in conjunction with the increased lung damage so that lung function may be compromised independently of vascular leak. Another possibility is that additional organs are impacted by the coinfection. Although there is no evidence of systemic spread of either pathogen (data not shown), there could be a systemic effect that compromises organ function. In addition, although we see no evidence of overwhelming pathogen burden at early timepoints post-coinfection, it is also possible that there is a loss of control of either the virus or the bacteria at later timepoints, in some mice, which may contribute to the death of these coinfected animals. This seems unlikely though considering that the majority of mice that succumb to infection do so within the first 3 days post-coinfection where we observed no evidence of overwhelming pathogen burdens.

While other models of coinfection point to major perturbations to either host resistance or tolerance as the cause of increased mortality, IAV/*K. oxytoca* coinfection is instead characterized by more subtle alterations in both of these host responses that work synergistically to decrease survival. These data, in conjunction with previously published studies, demonstrate that the impact of IAV infection on host resistance and tolerance responses is dependent on the bacteria that comprises the secondary infection. Although we observed similar trends as have been previously reported in other coinfections, our data demonstrate that even slight dysfunction of these host responses can lead to poor disease outcomes and it is likely that there are other mechanisms of both host resistance and tolerance that factor into determining the outcome of coinfection. Understanding how IAV impacts the response to a secondary bacterial infection is crucial in producing more effective treatments for these complex pulmonary infections.

## Materials and methods

### Nasopharyngeal swab sampling

The survey study was approved by the IRB (institutional review board) at Memorial Hospital of RI before any samples were obtained. The study samples were residual, spent, clinical samples of nasopharyngeal washings obtained in 0.9% normal saline from patients with influenza-like illnesses. These patient samples were obtained through the emergency room and acute care outpatient clinics during the influenza season of 2013–2014. The clinical laboratory performed rapid diagnostic antigen detection methods and by standard PCR methodologies for influenza virus. The research samples were obtained from spent samples prior to their final disposal. A waiver of informed consent by IRB approval was granted as the samples were patient de-identified by the clinical laboratory staff before providing the samples for the research study. The samples were maintained at −80C until further study.

### Mice

All animal studies were approved by the Brown University Institutional Animal Care and Use Committee and carried out in accordance with the Guide for the Care and Use of Animals of the National Institutes of Health. The University is accredited by the Association for Assessment and Accreditation of Laboratory Animal Care International (AAALAC). Brown University's PHS Assurance Number: D16-00183 (A3284-01), expiration date July 31, 2022. The USDA Registration Number is 15-R-0003. Brown University IACUC was approved on September 28, 2016, and the animal protocol number is 1308000011. C57BL/6J mice were purchased from The Jackson Laboratory. Mice used were female and 7–9 weeks old.

### Pulmonary infection

Mice under anesthesia and analgesia by ketamine (70–100 mg/kg) and xylazine (20–40 mg/kg) injection were administered IAV intranasally in a volume of 30 μL using a sterile saline vehicle. Mice were infected with 300 PFU influenza A virus (A/WSN/33 (H1N1)) strain. Influenza A virus was obtained from Akiko Iwasaki at Yale University. It was propagated using MDCK cells using standard procedures. *Klebsiella oxytoca* was cultured from glycerol bead stocks in 50 mL Todd-Hewitt broth overnight. The next day 1 mL of the overnight culture was diluted in 50 mL Todd-Hewitt broth and allowed to grow to log phase before being washed and resuspended in a sterile saline vehicle. Mice were infected intranasally with 10^6^ CFU in a volume of 30 μL. Coinfected mice were administered 300 PFU IAV followed by 10^6^ CFU *K. oxytoca* 5 days later. Mice were monitored daily for a minimum of 3 days, and every other day for the remainder of the experiment, except for survival and weight monitoring which was conducted daily.

### Confirming klebsiella oxytoca identity

Bacterial DNA was isolated from a culture grown as described above using the QIAamp UCP Pathogen Mini Kit (Qiagen). Genomic DNA was sonicated to a median size of 300 bp using a Covaris S220 instrument. Fragmented DNA was subsequently prepared into sequencing libraries using the Ovation Ultralow Library System V2 from Nugen according to the manufacturer's instructions. The library was then sequenced on an Illumina HiSeqX machine in the 2x150 bp configuration, yielding a total of 3,234,751 paired end reads. Raw reads were deposited in the NCBI Short Read Archive (SRA) under accession number SRP148653. Reads were trimmed of Illumina adapters and low quality bases using Trimmomatic (67) and then assembled using SPAdes (version 3.11.0) (68). Preliminary Sanger sequencing of the 16S rRNA gene and Blastn analysis of individual contigs suggested that this strain was related to *Klebsiella oxytoca*. Therefore, we calculated average nucleotide identity of our scaffolds (ANI) based on MUMmer (69) using the web-based tool JSpeciesWS (70) using four completely sequenced genomes of *K. oxytoc*a as a reference. This analysis found that our strain, which we named JK01, was in fact *K. oxytoca* and shared >99% average nucleotide identity with strains CAV1335 and CAV1099. However, this strain was less similar to CAV1374 and KONIH1 (~92.6% ANI). This Whole Genome Shotgun project has been deposited at DDBJ/ENA/GenBank under the accession QMBO00000000. The version described in this paper is version QMBO01000000.

### BALF collection

Bronchoalveolar lavage fluid (BALF) was collected via exposing the trachea, inserting a BD Venflon IV catheter into the trachea, removing the needle and inserting a 1 mL syringe with PBS. 1mL of PBS was flushed into the lung and collected. BALF was then centrifuged to isolate cells which were counted on a Moxi Z Automated Cell Counter (Orflo) and used for flow cytometry analyses and cell-free supernatants were collected for cytokine analyses and albumin content quantification.

### Lung tissue cell collection

For isolation of cells from lungs, the right superior and middle lobes were perfused with 15 ml of PBS. The lung tissue was cut into small pieces and incubated for 60 min at 37°C in 2 ml of digestion media containing type 4 collagenase (Worthington Biochemical Corporation) and DNAse I (Sigma-Aldrich). Digested lung tissue was then sieved passed through a 70 μM cell strainer and washed with PBS. After washing the cell pellet was resuspended in 1.5 ml 44% Percoll/0.15M NaCl and layered over 1 ml of 56% Percoll/0.15M NaCl. Percoll layers were centrifuged at room temperature for 20 min at 600 g with minimal acceleration and deceleration to form a gradient with a band of cells at the interphase which were then collected and washed with 10 mL PBS. Isolated lung cells were counted on a Moxi Z Automated Cell Counter (Orflo) and used for flow cytometry analyses.

### BALF albumin content quantification

Cell-free supernatants taken from BALF were tested for the concentration of albumin using the BCG Albumin Assay Kit (Sigma Aldrich) according to the manufacturer's instructions, using a dilution series of albumin standard to determine a standard curve which was used to calculate measurements of albumin in each sample. Absorbances were measured for each sample at 620 nm on a SpectraMax® M3 Multi-Mode Microplate Reader (Molecular Devices) using SoftMax Pro 6.4 software.

### Viral quantification

Viral genome copies were measured using RNA isolated from the unperfused right inferior lobe using the ReliaPrep™ RNA Tissue Miniprep System according to the manufacturer's instructions (Promega). RNA then underwent PCR with random hexamers (Invitrogen). Viral genome copies from the cDNA were then measured via qPCR using the forward primer (5′-CATGGAATGGCTAAAGACAAGACC-3′), the reverse primer (5′-CCATTAAGGGCATTTTGGACA-3′), and the probe (5′-[6-FAM]TTTGTGCCCA[BHQ1a-Q]-3′) specific for the M gene of influenza A viruses. qPCR was run on a Roche LightCycler® 96 Real-Time PCR System and analyzed with the LightCycler® 96 software.

### Bacterial quantification

Unperfused superior lobes of mouse lungs were harvested and homogenized by a gentleMACS™ Dissociator (miltenyi Biotec) in 1mL of PBS. The homogenate was immediately serial diluted by 10-fold up to six times. 5 μL of each dilution were then plated on a sheep's blood agar plate per sample. The plates were then incubated under 37°C overnight and the colonies were counted as a measurement for the bacterial load in the infected lungs.

### Flow cytometry analysis of cell subsets

The following antibodies were used to identify cell subsets: Ly6C eFluor 450 (clone HK1.4, eBioscience), F4/80 eFluor 660 (clone BM8, eBioscience), CD11c Brilliant Violet™ 711 (clone N418, BioLegend), MHC II PerCP-eFluor 710 (clone M5/114.15.2, eBioscience), CD206 PE-Dazzle™ 594 (clone C068C2, BioLegend), TNFα Alexa Fluor® 488 (clone MP6-XT22, eBioscience), Siglec-F PE (clone E50-2440, BD Biosciences) and Ly6G PE/Cy7 (clone 1A8, BioLegend). Dead cells were excluded from analyses using Fixable Viability Dye eFluor 506 (eBioscience). For surface staining, cells were first washed with 1x PBS then incubated with Fixable Viability Dye diluted in 1x PBS for 20 min at room temperature. Cells were washed again with 1x PBS and then treated with anti-CD16/CD32 Fc receptor blocking antibody (clone 2.4G2) in 1x PBS (1% FBS) for 10 min on ice. Surface staining antibodies were then added and incubated for 30 min on ice. Cells were washed, then fixed and permeabilized with BD Cytofix/Cytoperm™ for 20 min on ice (BD Biosciences). Then cells were washed and stained with intracellular antibodies in the permeabilization buffer from the BD Cytofix/Cytoperm™ kit on ice for 30 min before undergoing a final wash and resuspension with 1x PBS (1% FBS). Samples were acquired on an Attune NxT Acoustic Focusing Cytometer (Thermo Fisher) or a FACSAria Flow Cytometer (BD Biosciences) and downstream analyses were performed using FlowJo v10 software (Tree Star, Inc.). Isotype, fluorescence minus one, and unstained samples were used as controls to determine positive and negative gating of experimental samples. Viable cells were determined by first gating out doublets and debris using forward and side scatter properties and then selecting for cells with low staining with Fixable Viability Dye eFluor 506 (Supplemental Figure 1A). Total cell numbers of each cell subset were determined by multiplying initial cell counts obtained on the Moxi Z Automated Cell Counter (Orflo) by the percentage of total viable cells.

### Cytokine analysis

Cytokine concentrations were determined in BALF using a custom LEGENDplex bead-based immunoassay (BioLegend) according to manufacturer instructions. The samples were acquired on an Attune NxT Acoustic Focusing Cytometer (Thermo Fisher) and the data files were analyzed by LEGENDPlex Data Analysis Software (Vigenetech). IL-6 concentrations in BALF were determined via the BD OptEIA™ ELISA set according to manufacturer instructions (BD Biosciences).

### LDH cytotoxicity assay

The LDH cytotoxicity assay was conducted using the Pierce™ LDH Cytotoxicity Assay Kit according to the manufacturer's instructions on BALF samples from days 1 and 3 post-coinfection. Absorbances were measured for each sample at 490 nm and 680 nm on a SpectraMax® M3 Multi-Mode Microplate Reader (Molecular Devices) using SoftMax Pro 6.4 software.

### Statistical analysis

Statistical analysis was performed using GraphPad Prism software. Fisher's exact test, log rank, ANOVA followed by Tukey's multiple comparison tests, and *t*-tests were performed where appropriate.

## Author contributions

KL and AJ contributed to the conception and design of the study; KL performed the animal experiments, analyzed the data, performed statistical analysis, and made the figures; JM-L, DC, and PB identified and sequenced the *K. oxytoca* strain; SO obtained and provided the nasopharyngeal patient samples; KL and AJ wrote the manuscript; All authors contributed to manuscript revision, read and approved the submitted version.

### Conflict of interest statement

The authors declare that the research was conducted in the absence of any commercial or financial relationships that could be construed as a potential conflict of interest.

## Supplementary material

Supplemental Figure 1 Flow cytometry gating strategy. First, viable cells were determined by gating out doublet cells based on FSC-A/FSC-H and SSC-A/SSC-H and then cellular debris/dead cells were gated out on FSC-A/SSC-A. From there, viable cells were gated on their low staining of viability dye **(A)**. Viable cells were further separated into cell subsets **(B)**. First, viable cells were gated based on Ly6G and F4/80. Ly6G^+^F4/80^−^ cells were called neutrophils. Ly6G^−^F4/80^+^ cells were further gated on CD11c. Ly6G^−^F4/80^+^CD11c^+^ cells were called alveolar and repopulating macrophages. Ly6G^−^F4/80^+^CD11c^−^ were further separated based on expression of Ly6C and MHC II. Ly6G^−^F4/80^+^CD11c^−^Ly6C^+^MHC II^−^ cells were called inflammatory monocytes and Ly6G^−^F4/80^+^CD11c^−^Ly6C^+^MHC II^+^ were called infiltrating macrophages. Ly6G^−^F4/80^+^CD11c^+^ macrophages and Ly6G^−^F4/80^−^CD11c^−^Ly6C^+^MHC II^+^ macrophages were further separated by their expression of CD206 and MHC II or TNFα in order to determine polarization. To establish the identity of Ly6G^−^F4/80^+^CD11c^+^ cells, we compared their expression of Siglec-F, a known alveolar macrophage marker, and CD11c on days 1 (**C**) and 3 (**D**) post-coinfection in the BALF.

Supplemental Figure 2 An LDH assay was conducted on BALF from days 1 and 3 post-coinfection as a measure of cytotoxicity in the lungs. ^*^ denotes *P* ≤ 0.05 between indicated groups. Data were analyzed with ANOVA followed by Tukey's multiple comparison tests. Error bars represent SEM. Data are combined from at least four independent experiments with at least four mice per group.
