# Supplementary figures and images for: Coinfection With Influenza A Virus and Klebsiella oxytoca: An Underrecognized Impact on Host Resistance and Tolerance to Pulmonary Infections
Source: Front Immunol. 2018 Oct 29;9:2377. doi: 10.3389/fimmu.2018.02377 (PMC6217722; doi:10.3389/fimmu.2018.02377)

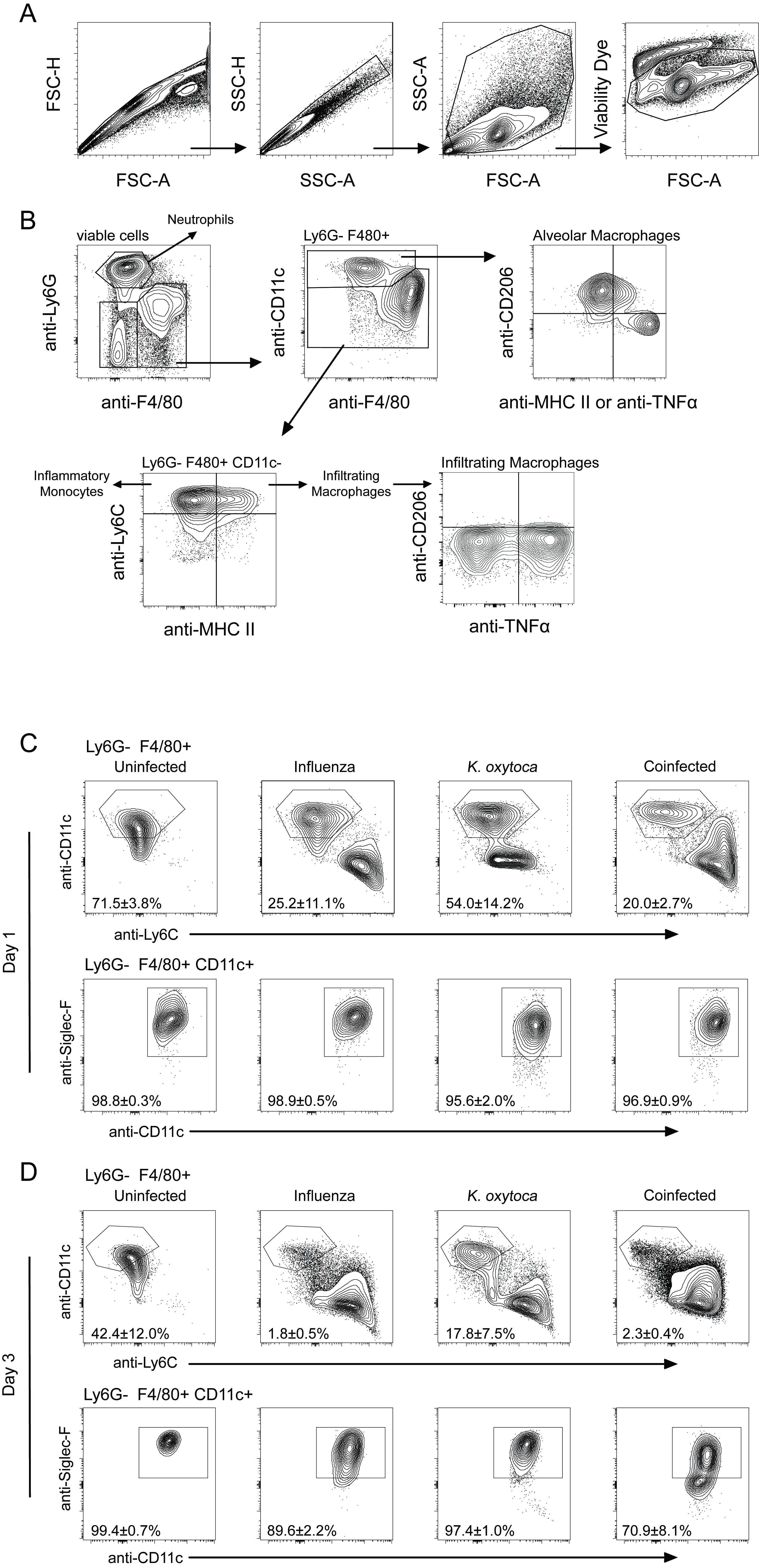

Supplement: Supplemental Figure 1 — Flow cytometry gating strategy. First, viable cells were determined by gating out doublet cells based on FSC-A/FSC-H and SSC-A/SSC-H and then cellular debris/dead cells were gated out on FSC-A/SSC-A. From there, viable cells were gated on their low staining of viability dye (A). Viable cells were further separated into cell subsets (B). First, viable cells were gated based on Ly6G and F4/80. Ly6G+F4/80− cells were called neutrophils. Ly6G−F4/80+ cells were further gated on CD11c. Ly6G−F4/80+CD11c+ cells were called alveolar and repopulating macrophages. Ly6G−F4/80+CD11c− were further separated based on expression of Ly6C and MHC II. Ly6G−F4/80+CD11c−Ly6C+MHC II− cells were called inflammatory monocytes and Ly6G−F4/80+CD11c−Ly6C+MHC II+ were called infiltrating macrophages. Ly6G−F4/80+CD11c+ macrophages and Ly6G−F4/80−CD11c−Ly6C+MHC II+ macrophages were further separated by their expression of CD206 and MHC II or TNFα in order to determine polarization. To establish the identity of Ly6G−F4/80+CD11c+ cells, we compared their expression of Siglec-F, a known alveolar macrophage marker, and CD11c on days 1 (C) and 3 (D) post-coinfection in the BALF. [file Image_1.tif]

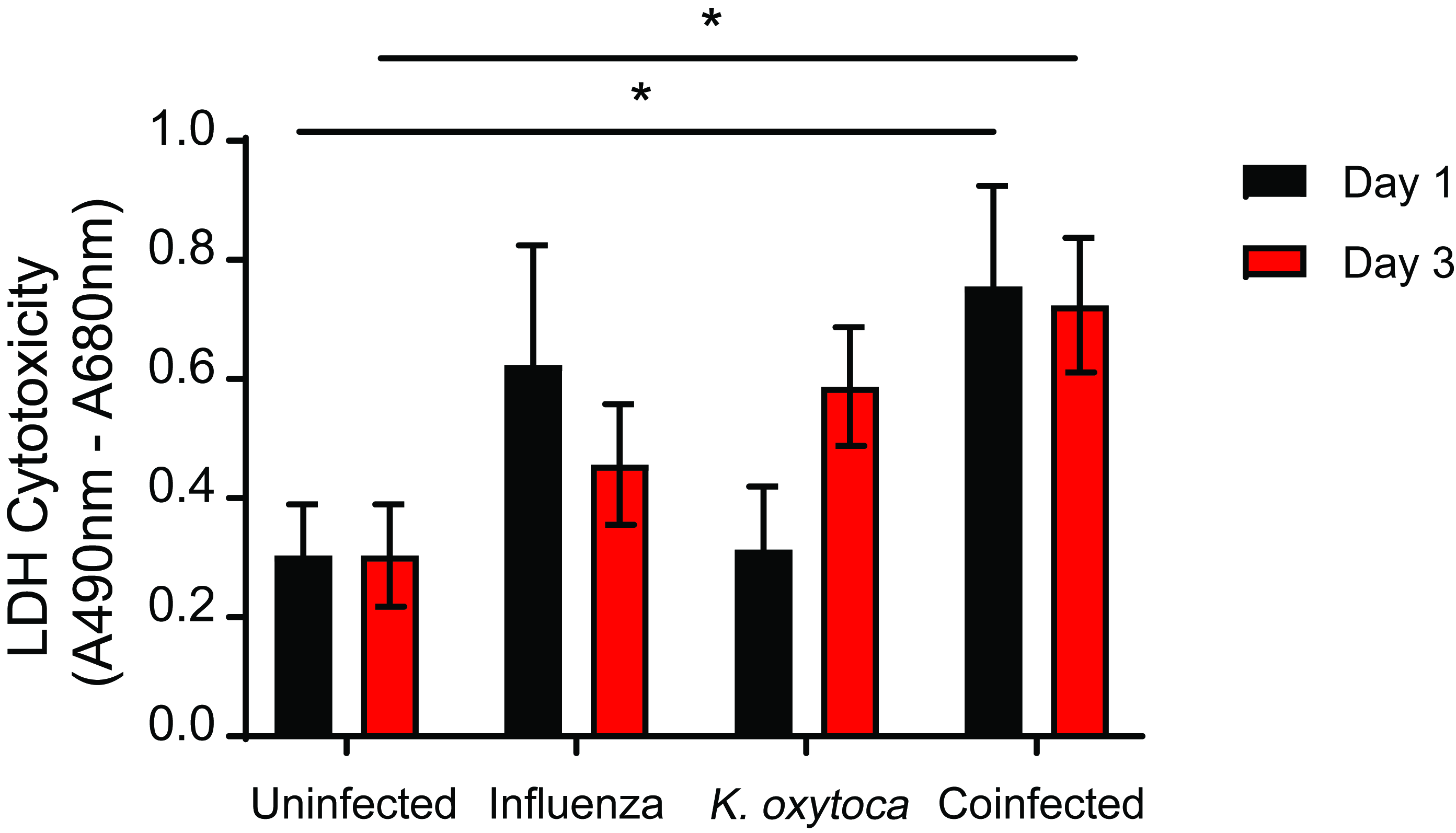

Supplement: Supplemental Figure 2 — An LDH assay was conducted on BALF from days 1 and 3 post-coinfection as a measure of cytotoxicity in the lungs. * denotes P ≤ 0.05 between indicated groups. Data were analyzed with ANOVA followed by Tukey's multiple comparison tests. Error bars represent SEM. Data are combined from at least four independent experiments with at least four mice per group. [file Image_2.tif]
